# Organ-wide 3D-imaging and topological analysis of the continuous microvascular network in a murine lymph node

**DOI:** 10.1038/srep16534

**Published:** 2015-11-16

**Authors:** Inken D. Kelch, Gib Bogle, Gregory B. Sands, Anthony R. J. Phillips, Ian J. LeGrice, P. Rod Dunbar

**Affiliations:** 1 Maurice Wilkins Centre, University of Auckland, Private Bag 92-019, Auckland 1142, New Zealand; 2 School of Biological Sciences, Faculty of Science, University of Auckland, Private Bag 92-019, Auckland 1142, New Zealand; 3 Auckland Bioengineering Institute, University of Auckland, Private Bag 92-019, Auckland 1142, New Zealand; 4 Department of Surgery, School of Medicine, Faculty of Medical and Health Sciences, University of Auckland, Private Bag 92-019, Auckland 1142, New Zealand; 5 Department of Physiology, School of Medical Sciences, Faculty of Medical and Health Sciences, University of Auckland, Private Bag 92-019, Auckland 1142, New Zealand

## Abstract

Understanding of the microvasculature has previously been limited by the lack of methods capable of capturing and modelling complete vascular networks. We used novel imaging and computational techniques to establish the topology of the entire blood vessel network of a murine lymph node, combining 63706 confocal images at 2 μm pixel resolution to cover a volume of 3.88 mm^3^. Detailed measurements including the distribution of vessel diameters, branch counts, and identification of voids were subsequently re-visualised in 3D revealing regional specialisation within the network. By focussing on critical immune microenvironments we quantified differences in their vascular topology. We further developed a morphology-based approach to identify High Endothelial Venules, key sites for lymphocyte extravasation. These data represent a comprehensive and continuous blood vessel network of an entire organ and provide benchmark measurements that will inform modelling of blood vessel networks as well as enable comparison of vascular topology in different organs.

Capturing the 3-dimensional (3D) organisation of complex tissues and organs, while crucial to understanding their biological function, represents a challenge for current microscopic imaging and analysis systems. A growing range of techniques now offer 3D imaging of tissues and even entire organisms at different scales and resolutions, each with particular advantages for specific research questions^1^,^2^. These imaging techniques are increasingly coupled to sophisticated image analysis tools as part of a trend towards extending microscopy as a quantitative rather than qualitative technique^3^. However, high-throughput and quantitative analysis of large 3D datasets is not trivial, and the paucity of powerful post-processing and 3D analysis tools for large image datasets has recently been described as a bottleneck in the field^4^,^5^.

There is a growing desire expressed in the literature for 3D descriptions of blood vessel networks to improve our understanding of their roles in tissue homeostasis and pathology, and enable computer modelling of their structure and function^6^,^7^. Studies aiming to capture extended 3D imagery of vascular channels have employed optic and non-optic volume imaging systems to gain comprehensive datasets^8^,^9^,^10^,^11^,^12^. However, these approaches have technical limitations, either due to the trade-off between resolution and scale, or due to distortions introduced during tissue processing. Microvascular networks therefore continue to be difficult to image at sufficient scale and resolution to make comprehensive measurements of network topology suitable for functional modelling.

Recently, a novel imaging technology has been developed which extends the scale of conventional confocal microscopy beyond its depth penetration limit, enabling high-resolution imaging over large tissue volumes. The ‘Extended-volume imaging system’ (EVIS) allows 3D imaging of several cubic millimetres of tissue at subcellular resolution (down to 0.4 μm)^13^. Given the scale and resolution provided by this imaging system, we sought to establish whether it could be applied to imaging and modelling of large microvascular networks. We therefore developed tissue staining and processing protocols that enabled 3D visualisation of murine microvasculature using EVIS, coupled with computational tools to enable topological measurements and re-visualisation of those vascular networks.

To capture a complete microvascular network we chose to image and model the vasculature within a murine lymph node (LN). These small organs are ideal as they have a single feed arteriole and corresponding vein, yet contain a highly complex vascular network with unique features. LNs have known substructures, and are compartmentalised into specialised cellular micro-domains to support immune responses by T and B lymphocytes, each supplied by intertwined channels of blood and lymphatic vasculature^14^,^15^,^16^. Specialised blood vessels with tall cuboidal endothelium, termed high endothelial venules (HEVs), are sites of mass lymphocyte trafficking, where most lymphocytes gain access from the circulation into the LN parenchyma^17^. The LN vasculature reconfigures extensively during immune activation, with blood endothelial cells proliferating and the feed arteriole remodelling within 2 days after immune challenge^18^,^19^. This vascular remodelling allows the LN to support lymphocyte accumulation, activation, and controlled expansion leading to LN swelling^20^,^21^. Since a functioning blood network is critical to LN homeostasis, it was recently discussed as a potential target for immune therapy^22^.

Here, we report high-resolution imaging of the blood vessel system of an entire murine LN and introduce computational procedures for processing and analysing those images in 3D. This combination of imaging and computation not only allows measurements of network topology, but also permits the re-visualisation of measurements in 3D, allowing the distributions of these parameters to be interpreted in a spatial context. We also show how these techniques can be used to compare the vasculature of functionally different LN subregions, and determine the contribution of morphologically distinct vessels such as HEVs to the entire network. This approach offers an innovative way of investigating the features of vascular networks that is now applicable to a range of different tissues.

## Results

**Figure 1:**
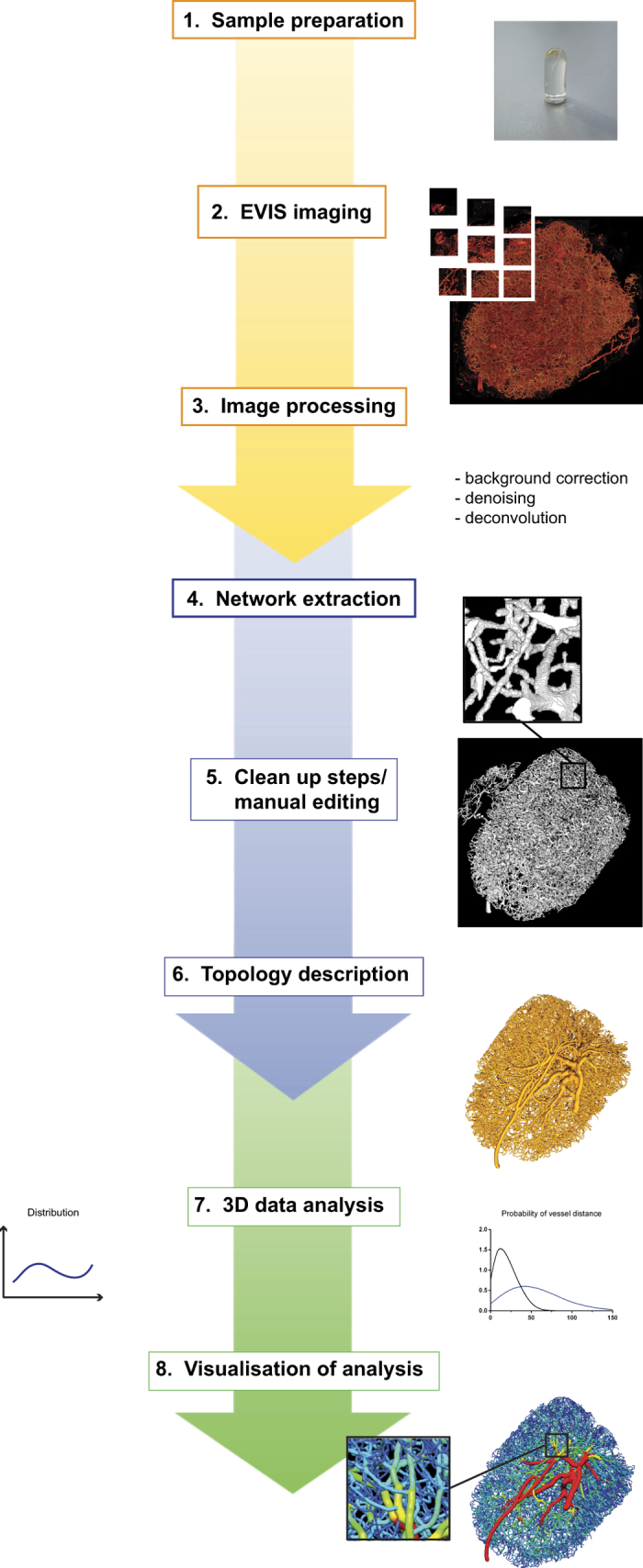
Workflow of 3D image acquisition and analysis. This graph outlines the working procedure used for EVIS imaging and computer-aided 3D analysis of LN blood vasculature. Firstly, the entire LN blood vasculature is stained by local perfusion with a fluorescent lectin and embedded in a stable resin to meet the prerequisites for seamless 3D imaging (1). Confocal images of the entire embedded tissue are acquired by EVIS, an iterative process of imaging and surface removal by an ultramill, and integrated into a 3D volume image (2). This procedure also involves purpose-designed image processing steps to remove optical noise and distortion (3). The generated 3D volume image can be utilised for extraction of the blood vascular network using a set of newly developed tools (4) and subjected to manual corrections using the 3D rendering program Amira (5) to subsequently provide a simplified version of the image data in form of a topology map (6). This map facilitates topological measurements in 3D (7) and further allows visualisation of the measurements in spatial context using 3D rendering software (8).

We employed EVIS imaging to capture the full blood vascular system of a LN and developed computer tools designed to aid the quantification of the resulting large dataset. Figure 1 summarises the methods we established and gives an outline of the workflow, including steps for vascular staining, sample preparation, imaging, image processing, network extraction, analysis, and visualisation. Details are provided in the Methods section.

### High resolution images reveal features of the lymph node vasculature

**Figure 2:**
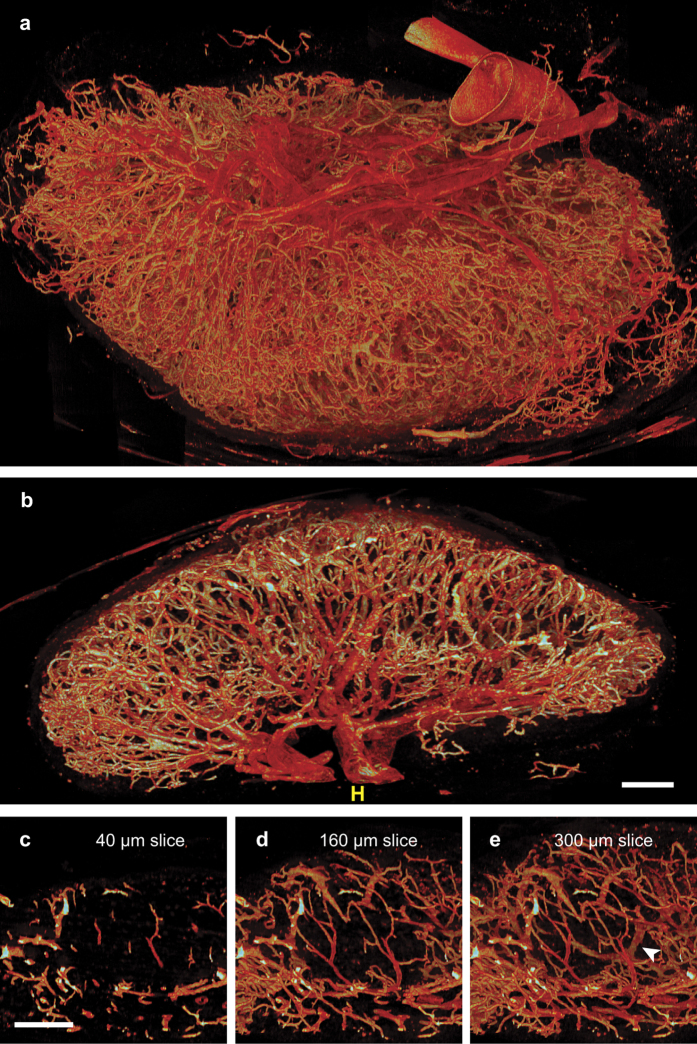
High-resolution 3D imagery reveals micro-anatomical features of LN vasculature. The entire vasculature of a murine mesenteric LN was fluorescently labelled and captured using EVIS imaging at a pixel resolution of 2 μm. The resulting 3D image comprised 63706 individual 500 × 500 μm images which resulted in a total image volume of 3.88 mm^3^ (1 GB). The dimensions of the imaged LN are 2503 × 1861 × 861 μm. The blood vasculature is visualised using the volume rendering program Voxx based on pixel intensity and colour coded as a heat map. The full volume view (**a**) and a thick optical section (**b**) display the overall organisation of the vasculature with dense capillary arcades and a clearly distinguishable hilum region (H) showing accumulation of large deeply red vessels. The 3D volume image allows zooming into regions with characteristic vasculature such as the B-cell follicles (**c**–**e**), which have an almost avascular core in thin volume slices (**c**) but reveal a surrounding network of vessels including a few vessels running through the centre in thicker volume sections (**d**,**e**). HEVs can be identified by means of their cobblestone morphology as a result of the luminal staining (arrowhead in **e**). Scale bars: 200 μm (**b**–**e**).

Using EVIS we acquired a comprehensive high-resolution volume image of the blood microvasculature of a mesenteric LN. 63706 individual 2D images were acquired and combined to give a 3D image with a voxel resolution of 2 μm, totalling approximately 1.5 GB. Viewed using voxel-based rendering software these 2-dimensional (2D) images show the extensive vasculature in fine detail (Fig. 2), with obvious regional differences in vascular composition. Most apparent is the accumulation of large vessels near the hilum, where the main artery and vein enter the LN while the outer surface comprises loops of smaller vessels (Fig. 2a,b).

In a form of ‘virtual histology’ it is possible to isolate and zoom into regions of interest within this volume image, for example the B-cell follicles (Fig. 2c–e). The follicles are characterised by sparse vascularisation in the centre and can seem almost avascular in thin sections (Fig. 2c) and vascular casts^14^,^23^. By selecting subvolumes of appropriate depth (160 to 300 μm), the dense vascular network surrounding follicles including HEVs (arrowhead in e) becomes evident. In this image three slim vessels can be followed passing though the centre of the follicle (Fig. 2d,e).

### Topological parameters of the extracted blood vascular network

**Figure 3:**
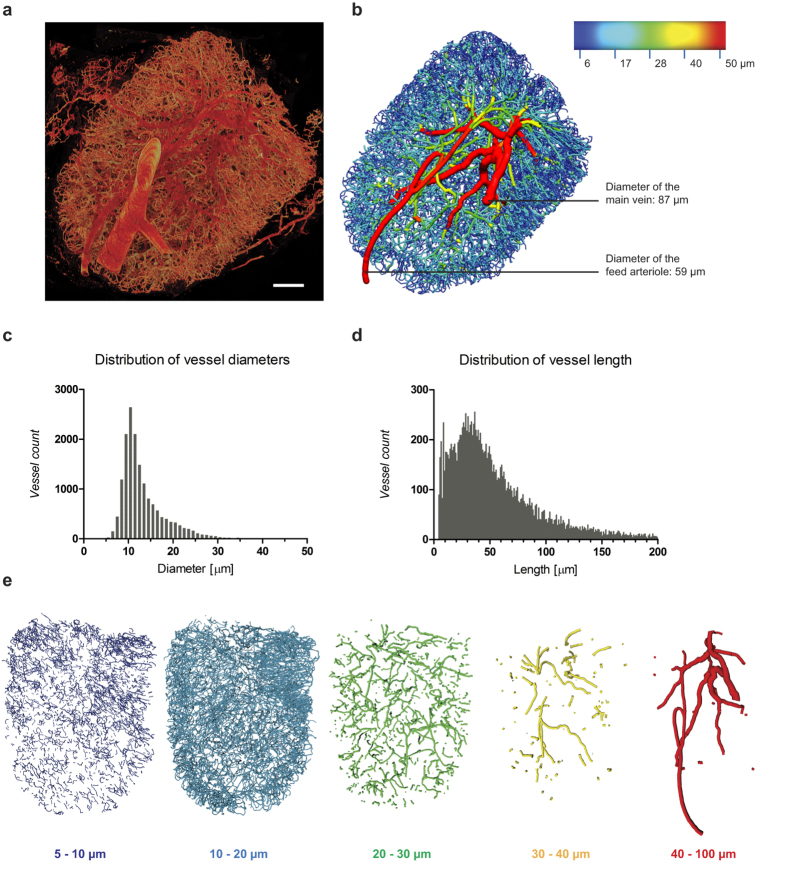
Topology analysis of 3D LN vasculature. Based on 3D imagery of full LN blood vasculature (**a**), the vascular network was extracted using a set of custom-developed tools and transferred into a numerical computer representation (**b**). The resulting 3D topology map allows detailed measurements of vascular network parameters such as the distribution of vessel diameters (**c**) and lengths (**d**). Both distributions are shown as a histogram display with a bin size of 1 μm (**c,d**). The diameter distribution within the network can further be visualised as a rainbow colour spectrum in CMGUI (**b**, Supplementary Movie 1) or sectioned in distinct ranges of 10 μm and colour-coded using Amira (**e**, Supplementary Movie 2). Thereby, the main feeding vessels become distinguishable and can be attributed as the main arteriole and vein, based on their diameters and morphology (**b**). Scale bar: 300 μm.

After applying a sequence of image-processing steps to extract the vessel network as a whole, the network was translated into a geometrical representation as a collection of tubes together with their connectivity, enabling measurement of network features (summarised in Table 1). A total of 16336 vessel segments and 12561 node points were identified within the network, comprising a volume of 0.171 mm^3^ representing 7.39% of the total LN volume of 2.31 mm^3^. Vessel diameters ranged from 4 to 87 μm, with an average of 13.47 μm, and the vast majority of diameters (97%) between 5 and 30 μm (Fig. 3). The feeding artery could be distinguished by its smooth appearance, diameter (59 μm), and homogeneous staining characteristics, compared with the rather lumpy appearance of the larger diameter (87 μm) main exiting vein (Fig. 3b). These results indicate that the blood vessel model adequately represents the full vasculature of the organ, including capillaries that are known to have diameters of 4–9 μm in the murine LN^24^. The majority of vessel lengths in the network are distributed between 5 and 200 μm (98%), with an average of 57 μm, and a combined length of 90 cm.

To provide further insight we sought to re-visualise important parameters within the 3D network model. Individual diameters of all vessels were represented as a colour spectrum across the whole network (Fig. 3b, Supplementary Movie 1). Arteries and veins could now be visually tracked from their parent vessels based on their gradated diameters, although the complexity of the background of the capillary bed hampered a clear vision of the arterial and venous trees (Fig. 3b). Therefore, to better distinguish the larger vessels within the network, we divided the network into distinct diameter ranges (Fig. 3e, Supplementary Movie 2). An accumulation of larger vessels can clearly be seen around the hilum, where the artery and vein branch into smaller daughter vessels. Overall the distribution of smaller vessels is relatively even across the main body of the LN but vessels with an intermediate diameter of 20–30 μm are located within an inner ellipsoidal region while a denser network of smaller vessels (with diameters of 5–20 μm) is abundant near the surface. This spatial distribution is consistent with the current understanding of LN vascularisation, where the main artery and vein enter the lymph node at the hilum, branch through the parenchyma as arterioles and venules, and form reconnecting capillary arcades below the capsule^14^,^23^.

### Branching of the 3D vascular network

**Figure 4:**
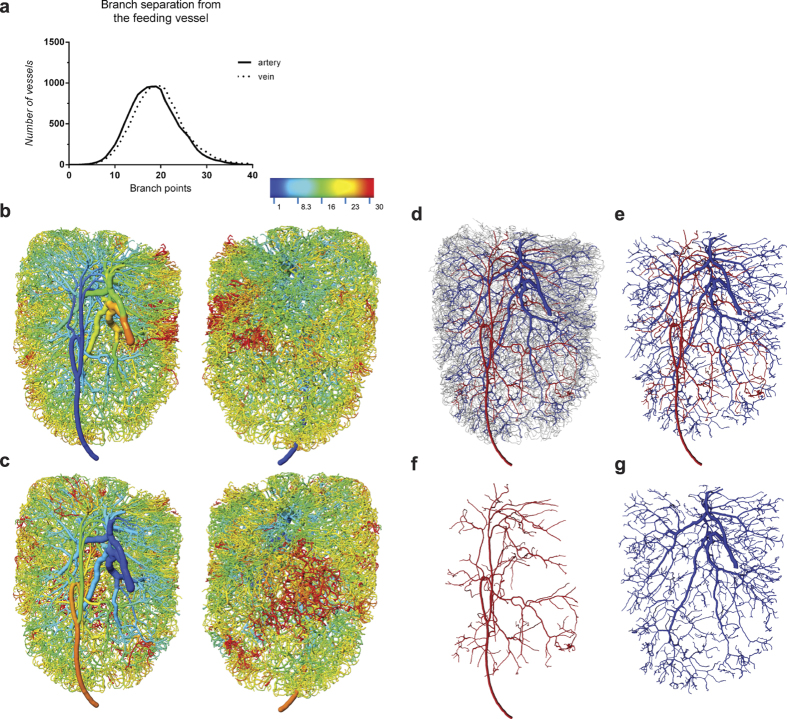
Network branching. The topology map of full LN vasculature was employed for 3D measurements on the branching pattern from feeding vessels and visualisation of the arterial and venous trees. The branch separation from the main arteriole and vein was calculated and plotted for all branching points within the network (**a**). Branching from the feeding vessel was further visualised in a rainbow spectrum display in CMGUI for the main arteriole (**b**) and the exiting vein (**c**), respectively (a full view is available in Supplementary Movie 3). By selecting only vessels with a diameter above 15 μm, abandoning smaller vessels including the capillary bed (grey), two connected networks can be easily separated, representing the arterial (red) and venous (blue) tree (**d**–**g**). Note that the arterial tree appears to have fewer vessels (**f**), since the diameters of arterioles are generally smaller than veins, and arterioles with a diameter below 15 μm are not included in this view. A 3D view of both vascular trees can be found in Supplementary Movie 4.

Based on our dataset it was now possible, for each node in the network, to identify the shortest paths to the feeding artery and to the main exit vein. The number of branches along each path was determined, providing branch counts from the main artery and the main vein for every node in the network (Fig. 4). Overall, the arterial and venous trees display very similar branching patterns. The distribution of branch counts from the artery ranges from 1 to 39, and 1 to 43 from the main vein; both branch counts show approximately Gaussian distribution and a peak around 18 branch points, with a mean of 20 (Fig. 4a). The range of branch counts was translated into a rainbow colour spectrum and the data displayed in 3D across the network (Fig. 4b,c; Supplementary Movie 3). The distribution reveals that the majority of vessels lie within 30 branch counts from the feeding artery, apart from a small proportion that lies further away (red in the spectrum display). The inner centre of the LN in close proximity to the hilum exhibits a low degree of branching for both the artery and vein (light blue areas). Remarkably, the rim of the LN displays a very homogeneous number of about 20–25 branches to the feeding artery (yellow areas; Fig. 4b). The distribution to the main vein is less homogeneous at the surface of the LN but contains a concentration of vessels in the centre that show a high branch count to the vein (Fig. 4c). In an alternative approach to visualising arterial and venous branches, we limited the displayed network to vessels with a diameter larger than 15 μm thus excluding the capillary bed and allowing us to isolate two large vascular segments representing the arterial and venous trees (Fig. 4d–g, Supplementary Movie 4). The separate views of the arterial and venous networks clearly illustrate the tissue coverage within this LN (Fig. 4f,g). The venous network of the chosen diameter range is very prominent in the LN and makes up about 44% of the total LN blood vessel volume, whereas the arterial tree within this diameter range represents only 14% of the vascular volume. Collectively, these data further reveal the value of displaying complex measurements in the 3D vascular model, adding new spatial information on vascular branching and arterio-venous relationships to the purely anatomical images.

### Distance from the nearest blood vessel

**Figure 5:**
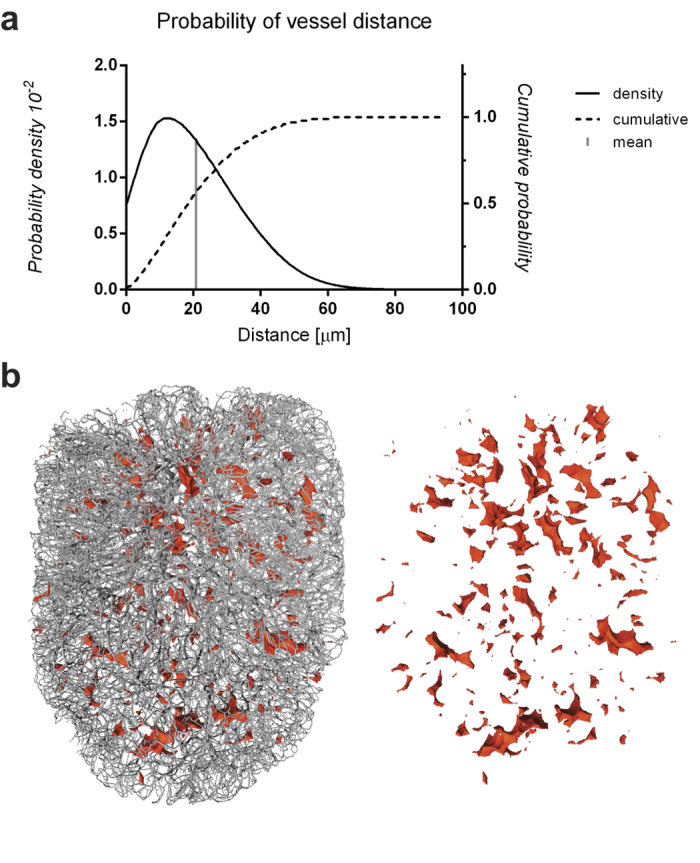
Tissue distance to the nearest vessel. To assess the distance from locations in the LN to the closest blood vessel, the LN volume was subdivided into a 4 μm grid and the minimum distance to the nearest vessel was calculated for about 34.8 × 10^6^ interior points (excluding points that fall within vessels) and plotted as a probability distribution (**a**). The average distance came to about 20.8 μm (grey line in **a**), while areas with a distance greater than 60 μm to a supplying vessel were visualised in red (**b**) to show voids within the vascular network (grey). Supplementary Movies 4 and 5 display the location of voids within the network model.

Given the fundamental role of the blood in maintaining cell function, we measured distances to the nearest blood vessel for all points within the LN to determine whether there were any relative “voids”^25^ in LN blood supply. The distribution of distances to the nearest vessel across the LN shows a peak around 12 μm and a mean of 20.8 μm (grey line, Fig. 5a); 99.3% of points in the LN lie within 60 μm of a blood vessel (Fig. 5a). Cells further than 60 μm from any vessel are potentially insufficiently supplied; therefore we created a 3D image of such locations. These “void” volumes were typically small and the majority accumulated around the hilum, where large vessels enter the LN but capillaries are rare (Fig. 5b, Supplementary Movies 4, 5). Because of the complexity of the network data, most of these voids could not have been detected visually, but became identifiable only with the help of 3D distance calculation tools.

### Identification and analysis of putative HEVs

**Figure 6:**
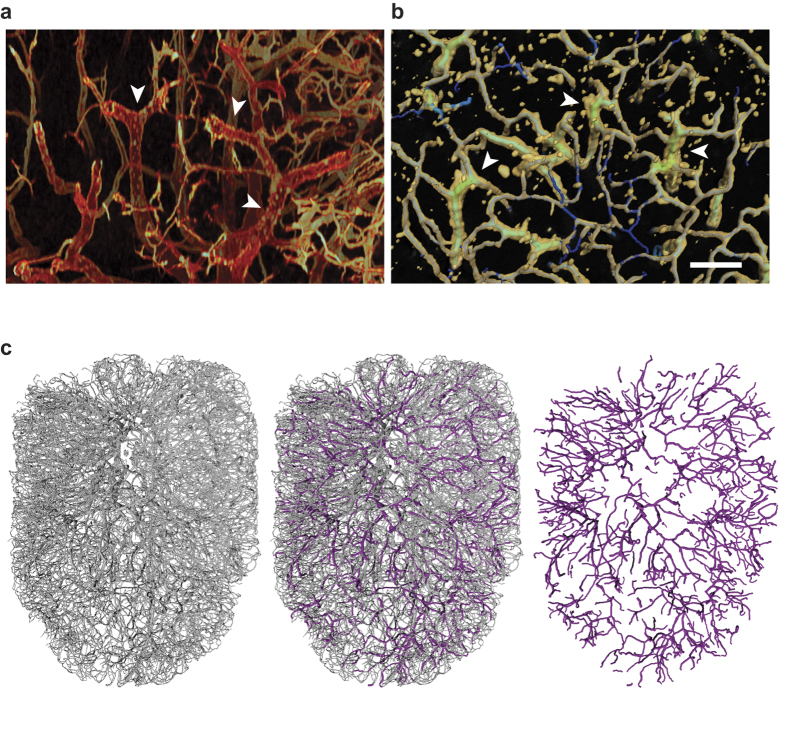
Identification and analysis of putative HEVs within the LN blood vessel network. Morphological parameters were used to extract HEV-type blood vessels from the vascular topology map for subsequent analysis and comparison with the overall vasculature. Within the 3D image of LN vasculature visualised in Voxx, HEVs can be identified by means of their cobblestone appearance (arrowheads, **a**). This phenotype is also evident in a surface reconstruction of the 3D image data in Amira and co-localises with vessels of intermediate diameter (green) of the extracted vascular topology map (arrowheads, **b**). This observation was used to extract putative HEVs (pHEVs, magenta) with a diameter between 16–32 μm from the vascular network (grey, **c**). Scale bar: 100 μm. The distribution of pHEVs within the vascular model can be traced in Supplementary Movie 4.

HEVs within the LN blood vasculature are the main site of extravasation for lymphocytes into the LN parenchyma. Previous studies using luminal blood vessel staining or vascular casting have demonstrated a so-called ‘cobblestone appearance’ within HEVs due to imprints of migrating lymphocytes on the endothelial surface^14^. Upon close examination we were able to identify such vessels in our 3D confocal LN vascular image. These vessels were evident from their bumpy endothelium in contrast to the smooth endothelium of other vessels and are visible in both intensity-based images (arrowheads, Fig. 6a) and surface representations (arrowheads, Fig. 6b). In an overlay with the network model we further noticed that the cobblestone morphology is associated with sudden diameter enlargements (Fig. 6b). These vessels had a diameter of 16–32 μm so vessels in this diameter range were selected from the full LN vascular network model, excluding small segments shorter than 40 μm as well as obvious arterial branches, giving a set of vessels with dimensions and locations within the network consistent with HEVs. These putative HEVs (“pHEVs”) were then overlaid with the LN vessel surface and vascular model to validate the selection criteria against the image data (Supplementary Figure 3). Key features of the pHEV network were quantified and compared to measurements on the full blood vascular network (Table 1). The pHEV network comprised 1107 individual segments with an average length of 107.5 μm and a total length of 11.4 cm. The average diameter in the pHEV network was, at 21.14 μm, 50% wider than the average LN blood vessel. Interestingly, the pHEVs made up about 12% of the overall vascular length but at 0.043 mm^3^ constituted 25% of the total blood vessel volume. The density of HEVs in the LN was, at 478 vessels/mm^3^, significantly lower than the density of all blood vessels which came to a count of 7057 vessels/mm^3^, yet they comprised a quarter of the total blood vessel volume. Overall, the pHEV network we extracted consisted of several connected subnetworks and a few isolated vessels (Supplementary Figure 3a) evenly interspersed across the full vascular network (Fig. 6c, Supplementary Movie 4).

### Blood vessel density in LN subregions

**Figure 7:**
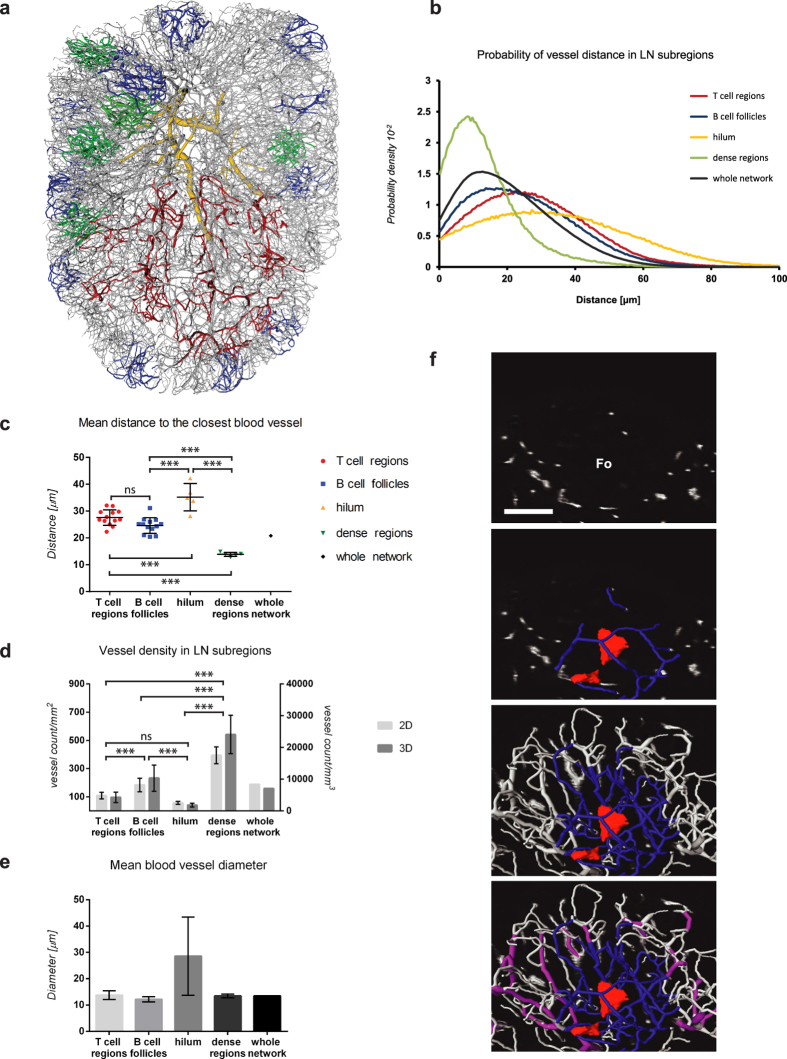
Comparative analysis of LN subregions. Based on regional differences in vessel density, functionally distinct LN subregions were isolated and evaluated. LN subnetworks colour-coded by location (red = predicted T-cell region, blue = B-cell follicles, yellow = hilum region, green = dense vasculature) are shown together with the whole vascular topology map (grey) in Amira (**a**). The average distribution of the distances to the closest blood vessel in each region reveals the characteristic vascular coverage in different parts of the LN (**b**). The mean blood vessel distance in LN subnetworks shows strong similarities between the predicted T- and B-cell regions, while being generally enlarged near the hilum, and notably shorter in dense regions (**c**). Vessel densities were evaluated using a 2D histology tool and 3D counts by the topology program, which both revealed similar proportions between LN subnetworks (**d**). With the exception of the hilum, which contains a range of very large and small vessels, the average diameter within LN subnetworks was nearly constant (**e**). A close-up of a B-cell follicle shows the avascular appearance in a 2D section, the selected vascular subnetwork (blue) enclosing a void with a distance greater than 60 μm from the nearest blood vessel (red), and the local pHEVs (magenta). Measurements of the mean and standard deviation (**b**–**e**) are representative of 13 individual 3D blocks (with a dimension of approximately 200 × 200 × 200 μm) from the predicted T- and the B-cell regions, and 5 blocks from the hilum and dense regions, respectively. Data were analysed using 1-way ANOVA, Tukey’s comparison. ***P < 0.001. ns = not significant, P > 0.05. Fo = follicle. Scale bar: 100 μm. The locations of analysied LN subregions together with avascular voids are visualised in Supplementary Movie 5.

We noticed clear regional differences in the vascular composition and density. Compared to locations at the LN border, the central region was characterised by more vessels with diameters of 20–30 μm, several voids with a distance greater than 60 μm to the nearest blood vessel, and a higher branch separation to the main vein. This region was found to correspond to the paracortical T-cell region of mesenteric LNs when investigated by 2D immunofluorescence microscopy (Supplementary Figure 2). As described above, regions with an almost avascular appearance located in the outer rim of the LN had typical morphological features of B-cell follicles. Based on these observations, we analysed LN subregions with visually discernible blood vascular morphology with respect to their diameter distribution, vessel distance, and vessel density. We selected 3D blocks from a central sphere (the predicted paracortical T-cell zone), the B-cell follicles (at the rim), and the hilum, and computed the distance to the nearest blood vessel (Fig. 7a,b). The predicted T- and B-cell regions show very similar distance distributions that are lower than those in the hilum, and slightly higher than the average distance within the whole network. This trend is also evident when the mean distances within subregions are compared (Fig. 7c). The equivalence of vessel distances in T and B cell regions initially seems counter-intuitive given the low density of vessels within the follicle centre. However, the analysis shown in Fig. 7 not only incorporates the follicular core but also the vasculature immediately surrounding follicles, which compensates for the high vessel distances that can be found within the centre of the follicles (Supplementary Figure 4). We also identified regions of dense vasculature outside these three defined zones, characterised by shorter distances to supplying blood vessels (Fig. 7a–c). To compare our 3D measurements with the conventional method for evaluating tissue sections, we created a 2D histology tool to evaluate vascular density in different compartments in 2D. This tool confirmed large density differences between subcompartments, and measurements of vascular density in 2D had the same hierarchy as in 3D (Fig. 7d). A combination of several different measurements that are feasible within the 3D model provides a new vision of the 3D topology of vessels within specific regions of the LN (Fig. 7f). Although B-cell follicles can appear nearly avascular in 2D images (Fig. 7f, upper panel), 3D visualisation shows the surrounding vascular loops supplying the follicle, as well as a central area within the follicle that is >60 μm distal from any of these vessels, which could be found in the majority of follicles in this study. Nearby HEVs that supply lymphocytes to the follicle can also be highlighted within the same image projections to provide a complete 3D view of the vascular environs (Fig. 7f) that is not evident in 2D views.

## Discussion

In this paper we present a new method to capture and analyse continuous vascular networks on an organ-wide scale and exemplify this procedure on the vasculature of an entire mouse LN. We used two innovative technologies: an automated confocal imaging system to generate high-resolution imagery of large tissue regions (spanning several mm); and novel custom-written computer tools that facilitated topological analysis of the large 3D image datasets using conventional workstations. This analysis includes measurements of structural features in 3D, such as branch counts from the feeding vessels to capillary beds, and distance evaluation over the entire network, and allowed re-visualisation of the parameters measured in a 3D context to enhance interpretation of the data. Since our method provides a higher level of detail than was previously possible, it helps to improve our understanding of the blood vessel topology in LNs and opens up new possibilities for similar large-scale anatomical studies of other organs. It also allows us to pose new questions regarding the organisation of structural compartments on a gross organ level and can help to define hallmarks of vascular pathology within them. The acquired information can now be used to advance computer models of immunity or vascular anatomy^26^ and inform large-scale modelling projects in system biology^27^,^28^.

## 3D capture of vascular networks on an organ-wide scale

Motivated in particular by research in developmental biology, oncology, and brain physiology, new imaging devices have emerged in the last decade to enable coverage of large tissue areas or even entire embryos^1^,^2^,^6^. Besides traditional non-optical macroscopic imaging approaches such as magnetic resonance imaging and X-ray computed tomography, extensive 3D information can now also be accumulated by mesoscopic techniques using optical systems with plane illumination or serial 2-photon imaging^29^. In particular, optical projection tomography (OPT) and selective plane illumination microscopy (SPIM) have found application in the study of LN anatomy and were able to quantify the number of HEVs and their relationship to B-cell follicles and DCs across whole LNs^30^,^31^. These techniques offer non-destructive imaging of whole organs at the cost of a lower resolution but require complete tissue clearing^32^.

In general, optical resolution is a major limiting factor when imaging is performed on large specimens of several millimetres^9^. As a consequence, most 3D imaging technologies either focussed on gross anatomical features over large regions or structural detail in limited regions of interest but were not able to comprehensively capture information of fine cellular structures like capillaries over large volumes^9^,^33^,^34^. EVIS provides a solution to this problem by overcoming the typical tissue penetration, field of view, and speed limitations of confocal imaging, thereby allowing its application in high-resolution 3D imaging over several millimetres of tissue^13^,^35^,^36^. It is related to episcopic imaging techniques like the imaging cryomicrotome^37^ and high-resolution episcopic microscopy (HREM)^38^, which perform imaging on the block-face of a stage-fixed sample and thereby facilitate image registration for the seamless assembly of the captured image stacks. Our approach offers the additional advantage of eliminating out-of-focus light via the confocal imaging process and can provide a higher image quality, thus augmenting the detection of delicate fluorescently labelled structures^39^. The high quality staining we achieved also helped generate a high signal-to-noise ratio, facilitating extraction of the blood vessel network.

A very recent innovative imaging technique performs imaging on just-cut tissue sections of 1 μm thickness and is able to capture the entire vascular system and fluorescent structures in murine brain in a process termed micro-optical sectioning tomography (MOST)^10^. In comparison to this and other techniques employing serial sectioning, our block-face imaging approach limits the danger of loss of information and distortion that can occur during the cutting process while still being able to resolve fine structural details. Within the field of these evolving 3D imaging technologies, each seeking to break the limitations of tissue penetration, resolution, speed, and cost, we show that EVIS offers a feasible approach to capture continuous blood vascular networks in a whole organ at an image quality sufficient to generate accurate computer models. Moreover, EVIS is able to detect multiple fluorescent signals, which will be used to map several labelled structures simultaneously in future studies.

However, shortcomings of this method include the requirement for robust post-mortem staining and resin embedding of the entire tissue, time-consuming imaging compared to non-microscopic imaging technologies (2 weeks of automated imaging for the current study), and the ultimate destruction of the sample during the imaging process. Nevertheless, ongoing optimisation of these techniques is improving the speed of the process, enabling comprehensive studies of larger organs in the mouse, as well as larger disease sites such as tumours.

### A new pipeline for image processing and analysis

In addition to the challenge of acquiring images over large volumes, the generated content- rich datasets require powerful post-processing and 3D analysis tools, a need which is currently met by few commercially available programs^40^,^41^. In fact, sophisticated 3D imaging studies are often restricted to laboratories that are able to develop or access specialised image analysis software^31^. Some generalist open-source 3D visualisation and analysis programs have been developed for ‘bioimage informatics’, but most remain focussed on specific biological areas such as cell segmentation and neuronal tracking^40^,^42^.

In order to efficiently describe and analyse the LN vascular network, the pipeline we developed used a combination of off-the-shelf commercial products (notably Amira), public domain software (e.g. Voxx) and custom-designed tools. These tools overcome previous limitations by breaking down computationally intensive processing steps into individual tasks, allowing them to be performed on conventional computers and permitting parameters to be selected for each step to suit different datasets. In particular, we focused on tools to extract continuous blood vessel networks after intra-luminal staining. This staining technique, while relatively straight-forward, can lead to apparent staining gaps in the blood vessel walls, so we developed a tool to fill these gaps by converting target voxels based on their ‘insideness’ within a vessel, solving a major bottleneck in extracting continuous networks from samples stained in this way. All the customised tools we developed could now be integrated into existing software packages for handling complex 3D data such as Vaa3D^5^,^42^.

An additional specific tool we developed enabled calculation of the distance to the nearest blood vessel across the entire tissue volume. This revealed only small “voids” with a distance greater than 60 μm to the nearest vessel, scattered predominantly near the hilum of the LN. While such measures are not by themselves sufficient to draw conclusions on the oxygenation of different tissue zones^43^, the relatively short blood vessel distances measured throughout our vascular model do not support the idea that there are hypoxic regions in the LN, at least in the non-reactive state. Such tools could also be used to identify and visualise “voids” in the vascular network in normal tissues or in pathological conditions where physical gaps in blood supply may have prognostic significance^44^. Combining all the parameters we measured in 3D (e.g. blood vessel diameters and lengths, along with network branching characteristics) might ultimately enable prediction of blood flow through different tissue regions^45^, at very high spatial resolution. Measurements on the scale we achieved may advance understanding of tissues known to develop hypoxic areas such as the retina^46^, or in disease situations where hypoxia is common^47^,^48^. Previously established computer models of vascular flow and drug transport are likely to benefit from the extensive blood vessel datasets these techniques can produce, with potential impact on design of therapeutic agents^43^,^49^.

### The microvasculature of lymph nodes

LNs have a fundamental role in the establishment of immune responses yet we lack a complete picture of their internal architecture^50^,^51^. We were motivated to develop techniques capable of comparing the vasculature in specialised LN subregions, and to enable future studies quantifying the dramatic vascular remodelling that takes place within LNs during an immune response. Previous electron microscopy studies of casts of blood vessel networks provided valuable insights into the overall arrangement of LN blood system at high resolution but only gained ‘snapshots’ of a selected point of view rather than truly 3D datasets^16^,^23^. More recent studies aiming to capture extended 3D imagery of LN vascular channels have employed serial sectioning^52^, and mesoscopic optical imaging techniques^30^,^31^, each with its own limitations, as discussed above.

Many of our observations are consistent with previously reported descriptions of the vascular organisation in LNs and the presence of an extensive venous network, as was previously suggested by Kowala and Schoefl^53^. But in addition, our data now provide precise measures of an entire vascular tree and the opportunity to view the vasculature from different optical angles, zoom into regions of interest, and compare structural parameters within them. A major improvement to previous studies is the precise 3D registration of the vascular network including capillaries, which in the murine LN are reported to have diameters of 4–9 μm^24^, and can be readily captured at the 2 μm voxel resolution that was used in this study. We acknowledge that precise vascular diameters are difficult to determine given the tissue shrinkage during embedding (20%), the resolution of the imaging (about 2 μm), the image thresholding applied (1–2 μm), and the transformation into a topology map (1%, see Supplementary Note 1). Nevertheless, processing parameters were chosen carefully to assure the match of processed images with the raw data and our measurements gain confirmation from the results of comparable imaging techniques^53^,^54^.

While the diameter measurements between ours and other LN data are largely consistent, the vascular volume fraction shows slight discrepancies between several studies, which can likely be attributed to tissue-specific differences, varying image resolution, and different methods being used for segmentation and vascular tracing. Our finding of a vascular volume of 7% is very similar to previous studies on heart (8–9%)^55^, muscle (9%)^25^, a tumour (8%)^8^, and a 2D study on LN sections (>6%)^56^, although significantly smaller fractional vascular volumes of 0.5–1.4% have been reported for the brain^10^.

An example of the utility of the complete network described here is the branch counts we were able to conduct over the entire network, based on the clear identification of a single feeding artery and exit vein in our dataset. We found the maximum branch separation between the major vessels and some capillaries to be in the order of 40, while the branch count between the artery and vein was noticeably lower. This asymmetry within the network could be attributed to uneven path lengths between arteries and veins (including some short loops) or to possible arterio-venous communications (‘shunts’)^14^. Identifying the origin of this asymmetry including the frequency and distribution of potential shunts will require further studies. Interestingly, it appears that the branch counts to the artery are more homogeneous across the network than the branch counts to the vein (Fig. 4b,c). This suggests that a higher selective pressure exists towards an even arterial supply of the tissue, which can be argued as being physiologically more important. Future studies will reveal whether this observation is consistent in other tissues.

In the context of LN function, HEVs are of high importance as key entry sites for lymphocyte into the tissue. We introduce a morphology-based approach to identify and extract putative HEVs from the vascular model, for subsequent analysis using our tool set. We acknowledge that this investigation has clear limitations – since we used a defined diameter range for selecting the pHEVs it is not possible to comment on the real diameter distribution. Nevertheless, we observe remarkable parallels to findings of other researchers who employed a common HEV marker and discovered similar HEV lengths and network distributions in peripheral LNs of comparable size^54^. Therefore, we believe that our method could be a valuable addition to vascular investigations in LNs in situations where applying an additional HEV maker is not feasible. Our comparison of the pHEV and the full blood vascular network also exemplifies a sophisticated 3D analysis of two qualitatively different networks and, to our knowledge, represents the first study of a complete vascular LN network in combination with HEVs.

Uniquely in this study, we combine all the measurements over the full LN blood vascular network to discriminate several zones with distinct topological features in their vascular supply. The rim of the LN shows a higher density of predominantly small vessels, surprisingly consistent branch counts of 20–25 to the main artery, and few voids. The predicted T- and B-cell regions show clear similarities in vascularisation, substantially higher than the sparsely vascularised hilum, but lower than the regions of dense vasculature found between the hilum and the rim. While additional stains for the lymphatic compartment will help identify these regions of dense vascularity more precisely, it is intriguing that these areas of potentially higher oxygen consumption are near the junctions of the paracortex with the medullary sinuses, sites of both active migration and interaction between lymphocytes and antigen-presenting cells^52^,^57^,^58^. The quantification of morphologically distinct zones within a continuous network is a critical advantage of our method over conventional histological or single-cell analysis.

In summary, we have established a new technique to comprehensively label, image and analyse microvasculature in 3D, based on simple intraluminal staining with a fluorescent lectin. For subsequent studies, we propose comparison of different blood vessel networks during homeostasis, inflammation, and disease, based on the multiple topological measurements enabled here. These studies will not only clarify the structure of microvascular networks in health and disease but will also improve modelling of phenomena such as hypoxia and drug distribution.

## Materials and Methods

### Workflow overview

EVIS was used to generate a comprehensive 3D image data set of the vasculature of a murine lymph node, and a combination of existing and new computer tools was used to model and quantify the resulting large network.

### Sample preparation

All experiments were undertaken with the approval of the University of Auckland’s Animal Ethics Committee and were in accord with its guidelines and the requirements of the New Zealand Animal Welfare Act. Extended-volume imaging requires a sample which is consistently stained throughout the whole tissue volume and stabilised by embedding in a synthetic resin. To achieve full tissue staining of the LN blood vasculature we established a local perfusion protocol utilising the fluorescent lectin wheat germ agglutinin (WGA), which was pumped through the abdominal aorta to label the mesenteric LNs via their arterial supply. More precisely, FVB/N mice (11 weeks old) were briefly anaesthetised with 5% isofluorane then killed by cervical dislocation. A laparotomy was rapidly performed and the lower abdominal aorta carefully exposed. The infra-renal aorta was incised and cannulated with a 23 gauge needle-barrel (Becton Dickinson, SG) mounted onto the end of catheter tubing (Critchley Tyco Electronics Pty limited, AU) which was attached to a syringe (Becton Dickinson, SG). The cannula was tied in place and the aorta was ligated just inferior to the diaphragm and the heart tissue disrupted in the chest to allow venous drainage. This arrangement ensured the intestines and mesentery were well perfused. The perfusion was undertaken using a Genie™ syringe infusion pump (Kent Scientific, US). After an initial rinse with phosphate buffered saline containing Heparin (100 units/mL; Mayne Pharma Limited, AU), the tissue was perfused in sequence of the following solutions: Firstly, the tissue was exposed to 2 mL 2.5% phosphomolybdic acid (Santa Cruz, US) and 2 mL 4% paraformaldehyde (PFA; ProSciTech, AU), each at a flow rate of 250 μL/min, followed by staining with 1.5 mL WGA-biotin (Vector laboratories, AU) and 2.5 mL streptavidin-Alexa Flour® 568 (Life Technologies, US) at a flow rate of 50 μL/min, and fixed with 2 mL 4% PFA at 50 μL/min. Phosphomolybdic acid was initially used in an attempt to dampen autofluorescence but was later removed from our protocol as it did not noticeably improve the sample quality. Since the mesenteric LN consists of multiple LN entities fused into one long chain, we carefully micro-dissected the first LN at the beginning of the chain by an incision at the invagination between the first two segments. Following excision, the first LN from the mesenteric LN chain was further fixed in 4% PFA with 3% sucrose (Sigma-Aldrich, US) at 4 °C overnight before embedding in a synthetic resin.

Following fixation samples were washed in PBS for 3 × 10 minutes and dehydrated through incubation in a series of 30%, 50%, 70%, 90%, 100% alcohol for 30 minutes each. Samples were infiltrated with LR white™ resin^59^ (hard grade; ProSciTech, AU) by incubation in 2:1 and 1:2 mix of ethanol:LR white™ for 1 hour each, followed by incubation in pure LR white™ for 3 hours, a resin change, and an additional overnight incubation. Heat curing was performed at 60 °C for 4 hours in gelatin moulds (ProSciTech, AU). This procedure was accompanied by a noticeable degree of tissue shrinkage, resulting in a diameter decrease of LNs of ≈20%. The overall shrinkage is likely to affect our 3D measurements to a similar extent as in previous electron microscopy studies using fixation and embedding in LR white resin.

### EVIS imaging & image processing

Imaging of a mesenteric LN embedded in LR white™ was performed on a custom-built automated confocal imaging system, referred to as EVIS^13^. In brief, the system consists of a confocal laser scanning microscope (TCS 4D CLSM, Leica Microsystems, DE), a milling device (Leica SP2600 ultramill, Leica Microsystems, DE), and a high-precision three-axis translation stage (Aerotech, US), all controlled by imaging software written in LabVIEW^TM^ (National Instruments, US). The sample was mounted on the translation stage which enables EVIS imaging with precise xyz registration of the imaged location. The murine mesenteric LN specimen was illuminated using an Omnichrome krypton/argon laser (Melles Griot, US) and images were acquired using a 20x water immersion lens (HC PL APO, 0.70 NA, Leica Microsystems, DE), 4x line averaging, and an image overlap of 50%. Each individual 8-bit (grayscale) image contained 256 × 256 pixels covering an area of 500 × 500 μm, which provides a pixel resolution of approximately 2 μm. Successive image places were acquired at an equivalent spacing of 2 μm (z resolution), resulting in an isotropic voxel size of (2 μm)^3^.

At this resolution it is assured that imaging can be performed in a timely fashion while still capturing fine vessels. In an iterative process, a 3D slice at the surface of the sample block is imaged, followed by the removal of most of the captured material by milling to allow additional imaging rounds of subjacent regions and subsequent reconstruction of a 3D image. This process maintains precise 3D registration of adjacent images, and avoids loss of information between sections compared to other 3D reconstruction techniques that rely on sample cutting prior to imaging^10^,^52^. Key to this process is the exact xyz-registration (with a precision of 0.3 μm) of a stably mounted sample and precise control of the surface removal by the ultramill in an automated process. In addition, purpose-designed image-processing software written in LabVIEW^TM^ is used to perform background correction on individual images, remove optical noise and distortion from assembled image stacks, and finally integrate overlapping image stacks into a 3D volume image^13^. To capture the vasculature of the mesenteric LN in this study, EVIS imaging was performed over 2 weeks leading to 63706 individual images covering an area of 3.8 mm^3^, which were subsequently combined to a 3D volume image of 1.5 GB (1550 × 1407 × 692 pixels).

Each individual image was background-corrected using a uniform reference image to compensate for uneven illumination of the sample. A wavelet-based denoising procedure was applied to individual image stacks to remove optical noise, followed by deconvolution employing the Richardson-Lucy algorithm which corrects for optical blurring. Once corrected, neighbouring image stacks were x-y aligned using cross-correlation and assembled into x-y mosaics. The resulting stack mosaics were manually overlapped in the z direction and blended together to produce a seamless 3D image. The integrated 3D volume image can be conveniently viewed and manipulated in the real-time volume rendering program Voxx (http://www.indiana.edu/~voxx/), which also facilitates the generation of high quality movie files.

### Network extraction & manual editing

To facilitate quantitative investigation of the acquired dataset, the pixel-based image first had to be processed to extract the vessel network structure. The generated confocal 3D volume image was translated into a numerical network description, or ‘topology map’, through a series of image processing steps, each allowing parameters to be adjusted to optimise each step. Firstly, the greyscale 3D image was translated into binary by applying a local thresholding algorithm. Segmentation was completed by employing a novel 3D algorithm that identifies voxels inside a vessel, allowing gaps in the vessel walls to be closed and achieving vessel filling, followed by isolation of the largest connected vessel network. Using a skeletonisation algorithm, the centrelines of structures in the network were identified^60^. Based on information from the network skeleton and the segmented image, a tracing algorithm was employed to map the location, shape, and connectivity of all network segments. The resulting description of the network structure is referred to as ‘network topology’. This network map also allows for manual editing of its elements which can be performed in 3D rendering software such as Amira, to remove adjacent vessels that do not belong to the main network of interest and to delete arterial branches from the selection of pHEVs. Manual editing was also performed to separate large vessels in close proximity within the hilar region.

A detailed descriptions of the tools used to transform the 3D volume image into a topology map is provided in the supplementary material (Supplementary Note 1) and the employed algorithms are available for download at GitHub (https://github.com/gibbogle/vessel-tools).

### Topology description & 3D data analysis

Once the network topology has been generated it is possible to derive statistical measures of vessel lengths and diameters, the numbers of segments within the network, and its vascular volume. We further developed tools to perform 3D analysis on the blood vessel network on the basis of this topology map. For example, we were interested in calculating the distances from cells in the LN to the closest blood vessel. Tools were created first to delineate the region of image occupied by the LN^61^ and then to compute the probability distribution of distances to the closest blood vessel border. To accomplish this, the total LN volume was overlaid with a 4 μm grid and distances to the closest blood vessel were calculated for about 35 million interior points (excluding points that fall within vessels). The availability of a complete 3D description of the blood vasculature also enabled us to determine the degree of branching in the network. A tool was established to calculate the branch separation for every node in the network to a specified starting point, which can be plotted as a probability distribution. Several additional accessory tools were developed to facilitate handling and cropping large tiff files, transferring files into different formats and compress them, or selecting regions of interest and vessels of particular diameter from the topology network. The latter was used to extract vessels of a diameter range associated with the HEV phenotype from the main blood vascular network and allowed this subnetwork to be investigated separately. In a similar fashion, vessels above a threshold diameter of 15 μm were selected to enable discrimination of the arterial and venous trees. We used a separate cropping tool to define blocks of 100 × 100 × 100 pixels (approximately 200 μm in x, y, z) from 4 subregions in the LN and investigated the blood vessel parameters in 13 individual blocks from the predicted T- and the B-cell regions, respectively, and 5 blocks each for regions with dense vasculature and the hilum. To validate our 3D measurements against the large body of research that describes blood vessel densities in the 2D space we established a ‘histology tool’, allowing us to calculate the number of vessels intersecting with a single plane, their diameters, and density.

### Visualisation of analysis

Finally, it is advantageous to visualise these measurements in 3D space. The network map is written in Amira Spatialgraph (http://www.fei.com/software/amira-3d-for-life-sciences/) and CMGUI file formats (http://www.cmiss.org/cmgui) which can be visualised in these programs as 3D renditions (with OpenGL) consisting of cylinders and spheres, facilitating the creation of animations. In addition, specific network characteristics such as the distribution of vessel diameters can be visualised with a rainbow colour spectrum, for example showing the location of large versus small vessels in a 3D fashion. Moreover, the branch count can be related to colours, allowing the branch separation from all points in the network to a start node to be represented as a colour spectrum in 3D renditions in CMGUI. Similarly, for the distance calculation a specialised tool enabled points located more than a specified threshold distance from the nearest vessel to be transformed into white voxels in a 3D tiff file, which allowed them to be visualised in 3D. This way, vessel voids could be identified in 3D and viewed together with the vessel network. Thereby, the acquired measurements can be interpreted in spatial context.

## Additional Information

**How to cite this article**: Kelch, I. D. *et al.* Organ-wide 3D-imaging and topological analysis of the continuous microvascular network in a murine lymph node. *Sci. Rep.* **5**, 16534; doi: 10.1038/srep16534 (2015).

## Supplementary Material

Supplementary Movie 1

Supplementary Movie 2

Supplementary Movie 3

Supplementary Movie 4

Supplementary Movie 5

Supplementary Information

## Figures and Tables

**Table 1:** Network parameters of pHEV^1^ and the total blood vessel network.

	Total bloodvessel network	pHEV network	pHEV fractionof the total bloodvessel network
Number of segments	16336	1107	7%
Number of nodes	12561	1256	10%
Mean segment diameter	13.47 μm	21.14 μm	157%
Mean segment length	57 μm	107.5 μm	187%
Network length	90 cm	11.4 cm	12.6%
Network volume	0.171 mm^3^	0.043 mm^3^	25%
Mean distance to the nearest blood vessel	20.8 μm	57.2 μm	
Fraction of the total LN volume	7.39%	1.86%	
Vessel Density	7057 vessels/mm3	478 vessels/mm3	

^1^pHEV = putative high endothelial venules.
