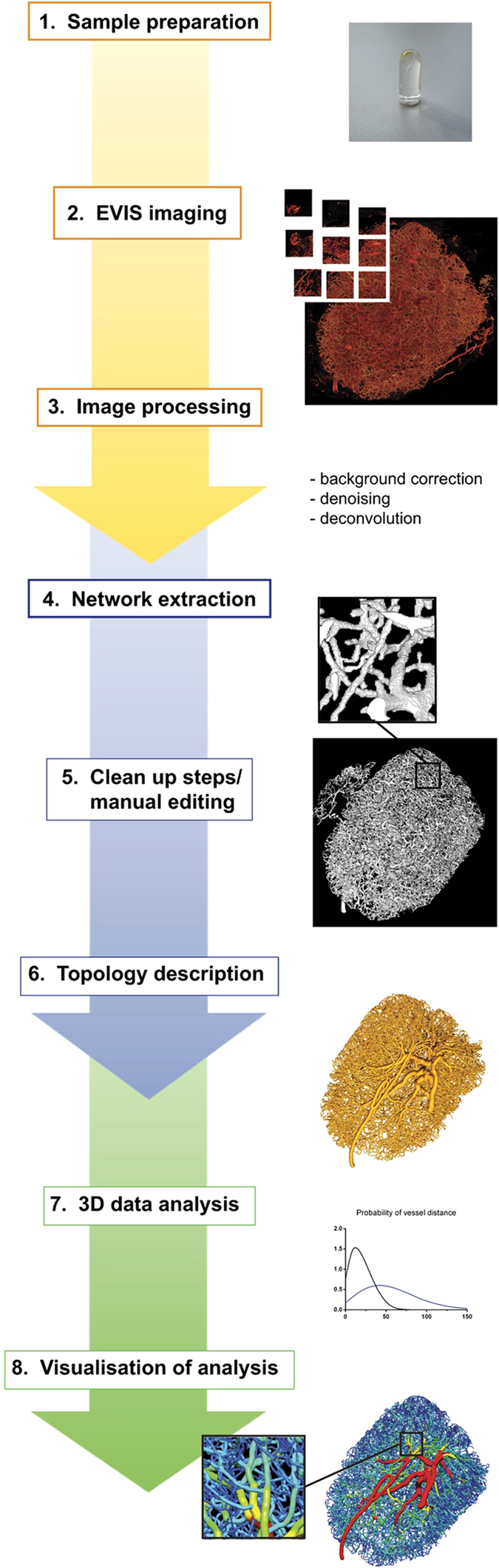# Corrigendum: Organ-wide 3D-imaging and topological analysis of the continuous microvascular network in a murine lymph node

**DOI:** 10.1038/srep20294

**Published:** 2016-02-04

**Authors:** Inken D. Kelch, Gib Bogle, Gregory B. Sands, Anthony R. J. Phillips, Ian J. LeGrice, P. Rod Dunbar

Scientific Reports 5: Article number: 1653410.1038/srep16534; published online: 11162015; updated: 02042016

This Article contains an error in Fig. 1 where the graph entitled ‘Distribution’ should have been omitted. The correct Fig. 1 appears below.

## Figures and Tables

**Figure 1:**