# Supplementary material for: Organ-wide 3D-imaging and topological analysis of the continuous microvascular network in a murine lymph node
Source: Sci Rep. 2015 Nov 16;5:16534. doi: 10.1038/srep16534 (PMC4645097; doi:10.1038/srep16534)
Supplement: Supplementary Information [file srep16534-s6.pdf]

SUPPLEMENTARY:

TITLE:

Organ-wide 3D-imaging and topological analysis of the continuous microvascular network in a murine lymph node

AUTHORS:

Inken D. Kelch[1,2]
Gib Bogle[1,3]
Gregory B. Sands[3]
Anthony R. J. Phillips[1,2,4]
Ian J. LeGrice[3,5]
P. Rod Dunbar[1,2]*

Affiliations:

1. Maurice Wilkins Centre, University of Auckland, Private Bag 92-019, Auckland 1142, New Zealand

2. School of Biological Sciences, Faculty of Science, University of Auckland, Private Bag 92-019, Auckland 1142, New Zealand

3. Auckland Bioengineering Institute, University of Auckland, Private Bag 92-019, Auckland 1142, New Zealand

4. Department of Surgery, School of Medicine, Faculty of Medical and Health Sciences, University of Auckland, Private Bag 92-019, Auckland 1142, New Zealand

5. Department of Physiology, School of Medical Sciences, Faculty of Medical and Health Sciences, University of Auckland, Private Bag 92-019, Auckland 1142, New Zealand

* Correspondence to: Rod Dunbar, r.dunbar@auckland.ac.nz

Supplementary Note 1: Detailed methodology for network extraction and analysis

The computational tools described in the following paragraphs are optimised to generate topological network descriptions from 3D image data of tubular structures such as the blood vasculature. They are available for download at GitHub (https://github.com/gibbogle/vessel-tools). If assistance is needed, please contact g.bogle@auckland.ac.nz.

A) Image processing and network extraction

The processing of the 3D greyscale image was carried out in two stages. The first stage is vessel segmentation: generating a binary image in which the voxels identified as falling inside a vessel are lit (intensity = 255) while those outside are dark (intensity=0). The second stage is network mapping: the network is extracted as a set of connected piece-wise linear tubular vessel segments. Use was made of the Insight Segmentation and Registration Toolkit (ITK), an open-source, cross-platform system that provides developers with an extensive suite of software tools for image analysis.[1,2] All the tools utilise ITK methods for reading and writing tiff image files, and certain ITK filters were also used – this is noted where applicable.

Stage 1 Image segmentation was the most challenging and most crucial task, and it required the development of several processing tools. The difficulty arises because of the nature of the staining process we used. The dye binds to endothelial cells in the vessel walls. If the resulting intensity had been at a uniformly high level in all vessels segmentation would have been relatively simple. Unfortunately, although on average the vessels were well-labelled, the intensity was highly variable and not always significantly higher than the background.

This resulted in "holes" in vessel walls and very faint labelling of some fine capillaries. The following procedure was followed to perform vessel segmentation.

Step 1 Local thresholding was used to improve the detection of faintly labelled vessels. The basic idea is that the threshold level depends on the average local intensity, a lower threshold being used when the local intensity is faint. Two parameters, T and D, determine how the threshold varies with average local intensity. To process a voxel at (x,y,z) with intensity V, the local intensity L is computed as the average over a small region centred on (x,y,z). If L > T-D the threshold level is T, otherwise the threshold is L+D. In other words, the threshold varies linearly over the range L < T-D, and is constant for L > T-D. The voxel is lit (set equal to 255) if V exceeds the threshold, otherwise it is set to 0. Typical parameter values are T=60 and D=3, giving the threshold function shown in Supplementary Figure 1.

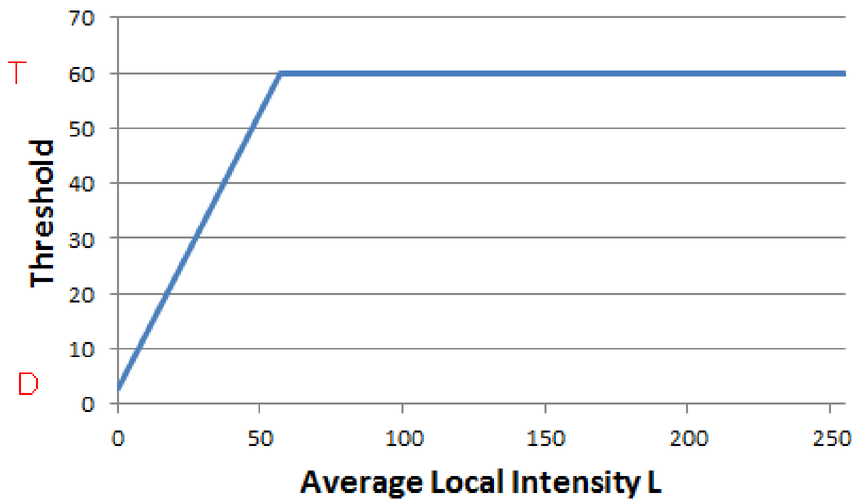

Supplementary Figure 1 | Variation of threshold with local intensity L.

This is for the parameter values T=60 and D = 3.

Step 2 In the binary image produced by local thresholding the vessels are mostly hollow and there are holes in the walls. To complete the segmentation it is necessary to fill the vessels, and since we were unable to locate anything suitable in the literature it was necessary to

develop a special algorithm for this purpose. The filling algorithm is based on the idea of a measure of "insideness" of a voxel – a way to quantify the degree to which a voxel appears to be within a vessel. The basic idea is to determine the distances from the candidate (dark) voxel to the nearest lit voxel in many different directions. It is convenient to use the 26 directions corresponding to the 26 neighbours of a point in a 3D rectangular grid. Voxels are located at regular intervals along these probe lines, with a spacing of 1, $\sqrt{2}$ or $\sqrt{3}$. The number of probes that encounter a lit voxel within a specified radius (one parameter of the method) is counted. If this count exceeds a threshold value (the other parameter) the voxel is taken to be inside, and is lit (set to 255). Typical parameter: maximum radius = 4, threshold count = 19. A list of candidate voxels is first created, recording those voxels that have a chance of being inside a vessel. This decision is based on a looser "insideness" criterion. The method is then applied in repeated sweeps through all the voxels in the candidate list, typically in four sweeps. To fill any remaining "bubbles" (completely enclosed voids) within the vessels, the ITK Binary FillholeImageFilter is applied.

Step 3 The final step of the segmentation is to isolate the largest connected object in the image. This is achieved using a program that finds all the "objects" in the image, where an object is a connected set of voxels. The largest such object corresponds to the vessel network, and it is saved as the final segmented vessel image. In the case of the LN data, the vessel network object was 99% of the lit voxels in the segmented image.

Stage 2 The segmented image of the vessel network is suitable for display purposes, but not immediately useful if quantitative measures of the network are required. Our ultimate aim is to quantify the changes in LN structure that are induced by infection, and for this we need a detailed numerical description of the network, conveying the dimensions and layout of the vessels, including their connectivity. As a convenient shorthand term, we refer to this description as the network topology. Once the network topology has been mapped it is

possible to derive statistics of vessel lengths and diameters, and, in combination with the segmented image, statistics of the distance to the nearest vessel. The sequence of steps is as follows:

Step 1 An ITK thinning (or skeletonisation) algorithm BinaryThinning ImageFilter3D[3,4] is applied to the binary image, producing a binary image containing only the lines of connected voxels, like beads on a string, that mark the centerlines of the vessels.

Step 2 A tracing algorithm is employed to extract the network structure from the skeleton image. This is the fundamental mapping step, resulting in a detailed description of the shape and connectivity of all the vessel segments. Each vessel is described by a set of 3D points, the locations of the centerline voxels. Data describing vessel connectivity and the locations of the junctions (where three or more vessels meet) is also generated – this information is required for graphical rendition (see later).

Step 3 The complex structure of the vascular network, in particular in regions where large vessels meet, provides a challenge to the thinning algorithm. Large vessels tend not to hold their shape through the sample preparation, and there are many flattened sections in the image. In such regions the thinning often does not produce the ideal skeleton – for example sometimes three vessels meet at the vertices of a tiny triangle instead of at a single point. Such processing artefacts require a "tidying-up" step. A variety of measures are employed to remove such obvious artefacts and also to "prune" very short dead-end vessel segments that are probably not physiological. The "tidying-up" of the network is carried out semi-automatically. The program Topo that traces the network automatically removes tiny triangles that are sometimes generated by the skeletonisation. These are triplets of centreline points immediately adjacent to each other, i.e. corresponding to adjacent voxels in the segmented image. These are clearly artefacts of the algorithm. Another measure applied

automatically in this program is "simplification". When there are more than five centreline points between two junctions (nodes), points are dropped in a way that ensures that no two points are closer than half the vessel diameter at that location. This smooths the vessel segment somewhat (reducing staircasing) and aids in diameter estimation. The Pruner program gives the user control over parameters that determine how the network is to be pruned – as the name suggests, this is the removal of "twigs" (vessels connected only at one end) with length/diameter less than a specified value.

Step 4 The numerical description of the network is completed by associating a diameter with each point along the vessel centerlines. At each point the area of the cross-section is estimated, and the equivalent diameter (the diameter of a circle with the same area) is recorded. The shape of vessel cross-sections varies widely, especially in the case of the larger vessels. To measure the cross-section it is first necessary to locate the plane it lies in. This requires estimating the direction of the centerline at the point – the cross-section plane is perpendicular to this. It is not sufficient to use the immediately adjacent points to define this direction, since such an estimate would be strongly affected by the voxel rasterisation. A smoothed estimate is obtained by basing it on two centerline points at least half a vessel diameter away on either side of the point. This requires first making a crude estimate of the area (and diameter), then using it to refine the definition of centreline direction at that point. Then working with the segmented image a number of probes equally spaced around 360 degrees (like spokes of a wheel) are used to find the distances from the centerline point (voxel) to the vessel boundary, i.e. to the dark voxels surrounding the vessel. The cross-section area is estimated from these distances.

The centerline points are very close together and the discreteness of the raster image inevitably lends a "staircase" appearance to the vessels. A smoother and more realistic-looking network is produced by dropping some points, using the criterion that points should

be no closer than 0.5 times the vessel diameter. Optionally an additional smoothing step can be applied, in which all the points on a vessel are given a diameter equal to the average diameter of the vessel. The average diameter is determined from the average cross-sectional area, defined as the vessel volume divided by vessel length. Inevitably the tissue sample includes some vessels external to the lymph node. These are removed using the Amira program (filament editor).

B) Network analysis

The resulting network map is represented in two ways, as an Amira Spatialgraph file and as a pair of CMGUI files (elements and nodes). The CMGUI files can be generated from the Spatialgraph file. Amira enables network viewing and editing, but CMGUI provides more flexibility in generating 3D renditions (with OpenGL), and facilitates the creation of animations. In CMGUI each element (each segment of a vessel) is rendered as a cylinder with a diameter that varies linearly along the length. Each node (each point along the vessel) is rendered as a sphere. The result is a seamless rendering of the network.

Several measures that characterise the network can be derived once the topology and the diameters have been determined. Probability distributions (actually histograms) of vessel length and vessel average diameter provide the most basic encapsulated description of the network. To provide a more accurate measure, weighting by vessel length is used to compute the diameter distribution. We were interested to determine the degree of branching in the network and developed a tool to calculate the branch separation for every node in the network to a specified starting point. From this the number of nodes on the shortest path between the starting point and another point in the network was counted. The code developed for this purpose employs Dijkstra's shortest path algorithm.[5] The branch count for all points in the network can be plotted as a probability distribution and also related to colours, allowing the

branch count to a start node to be represented as a colour spectrum in 3D renditions in CMGUI. In cases where the main dispersing or collecting vessel of a network could be identified, the branch counts were used to visualise the arterial and venous trees.

Another network property of interest concerns the distance of a point within the lymph node from the nearest blood vessel. The first step in computing the vessel distance distribution is to generate the "hull" file for the vessel network. The purpose of the hull file is to be able to determine, for a given voxel location (x,y,z), if the location lies within the LN tissue region. First, a binary image is created in which voxels that fall within a vessel are lit. From this image another binary image (the convex hull image) is generated using an ITK module BinaryCloseParaImageFilter [6], that employs a 'rolling ball" algorithm. In this image all voxels that lie within the convex hull of the network are lit, i.e. effectively the image corresponds to LN tissue.

Next a regular rectangular 3D grid of points is defined, spanning the LN region. The data in the hull file is used to determine the subset of these grid points that are within the LN tissue region. The grid spacing is chosen to be larger than the voxel dimension (to limit computational requirements) but small enough to give a large number of points within the LN.

For each of the tissue grid points the distance to the nearest vessel is computed, using vessel segment geometry and diameter information. The distance that is calculated is to the vessel wall – in this computation grid points that lie within a vessel are excluded. The distance distribution is then determined from the set of minimum distances.

Supplementary Note 2: Histology of LN vasculature

To help identify LN compartments within the vascular network, we compared thin volume sections and a single slice from the 3D volume image with immunostained sections of a comparable mesenteric LN (Supplementary Figure 2). Immunohistochemistry was performed according to a 3-colour protocol established by Lloyd, et al.[7] using the following antibodies: LYVE1 (MAB2125, R&D Systems, US), biotinylated CD31 (553371, BD Pharmingen, US), collagen I (ab34710, Abcam, US), anti-rabbit goat IgG Alexa Fluor 488 (A11008, Molecular Probes, US), anti-rat goat IgG Alexa Fluor 555 (A21434, Molecular Probes, US), streptavidin Alexa Fluor 647 (S-21374, Molecular Probes, US).

The observation of sparse vascularisation in the centre of the LN volume (Supplementary Figure 2 b, c) correlates with an equivalent density of $CD31^+$ vasculature in the paracortical T-cell area of a mesenteric LN (Supplementary Figure 2 e, f), which can be identified by means of its characteristic collagen arrangement. The B-cell follicles are easily distinguishable by lack of collagen and sparse CD31 staining in the immunosections (Supplementary Figure 2 d, e), which relates to their appearance in the 3D vascular volume (Supplementary Figure 2 b, arrowheads). Additional immunolabelling against LYVE1 marks the lymphatic endothelium which in the mesenteric LN is mainly located at the rim (Supplementary Figure 2 f). In contrast, discrimination of the lymphatic compartment against other regions in the 3D blood vascular volume cannot convincingly be performed based merely on vascular density. The dual-staining with LYVE1 and CD31 also reveals that CD31 weakly labels the lymphatic vasculature (Supplementary Figure 2 f, small panels in 2 d), which makes it necessary to subtract the LYVE1 signal in order to gain a clearer picture of the distribution of blood vessels (large panel in Supplementary Figure 2 d). Vascular labelling by perfusion with WGA on the other hand, does not involve additional stain on lymphatic structures in the 3D images, and can therefore deliver valuable information about the vessel

density. Taken together, these data provide a comprehensive overview of the spatial arrangement of LN vasculature and allow detailed insights into subcompartments.

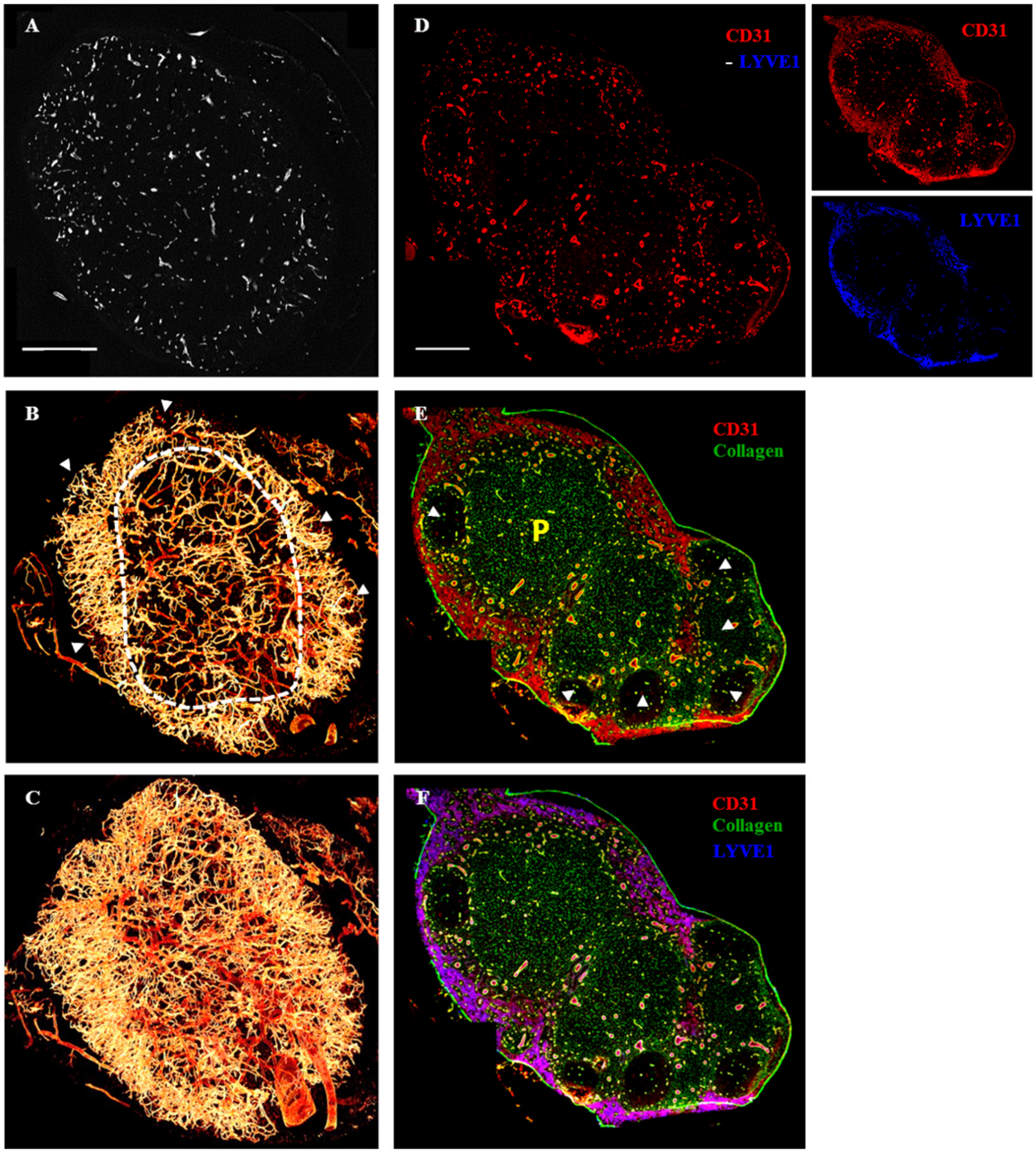

Supplementary Figure 2 │ Comparison of LN vasculature in 3D and 2D.

Comparison of fluorescent WGA-labelled LN blood vasculature (A, B, C) with immuno-stained LN cross sections (D, E, F) indicates similar vascular topology in subcompartments. An individual slice of the 3D vascular volume image (A) shows equivalent vessel density to an immuno-stained LN section labelled with the vascular marker anti-CD31 (D). Note that the CD31 antibody also stains the LYVE1+ lymphatic endothelium in addition to the blood vasculature (small panels in D). By subtracting the LYVE1 signal from the CD31 image using custom image processing software, the blood vasculature can be visualised by itself (large panel in D). Thick volume sections of the 3D vascular volume image generated in Voxx exhibit an ellipsoidal region of low vascular density in the centre (dashed line; B, C) which correlates with the LYVE1- and collagen+ paracortex ('P') in histology sections (E,F). B-cell follicles can be identified via sparse vascularisation (arrowheads, B, E) and lack of collagen stain (E). P = Paracortex. Scale bar: 500 µm.

Supplementary Note 3: Extraction of a putative HEV network

HEVs show characteristic morphological features such as rough ‘cobblestone’-like endothelium which we found to be accompanied by a vessel diameters of about 16-32 µm in the vascular model of a mesenteric LN. A selection of putative HEVs (pHEVs) was extracted from the vascular model based on these diameters (Supplementary Figure 3 a). In addition, small segments shorter than 40 µm and individual arterial vessels were excluded from the selection. To validate this selection against the raw image data, we superimposed the network model and pHEVs with a surface representation of the raw image using the 3D visualisation software Amira. The pHEV network was found to overlap well with rough endothelium in the surface representation (Supplementary Figure 3 g) and cover all vessels with increased diameters (shown in green) in the LN vascular model (Supplementary Figure 3 f). Thereby, the diameter-based selection of pHEVs was confirmed to match the morphological observation of HEVs.

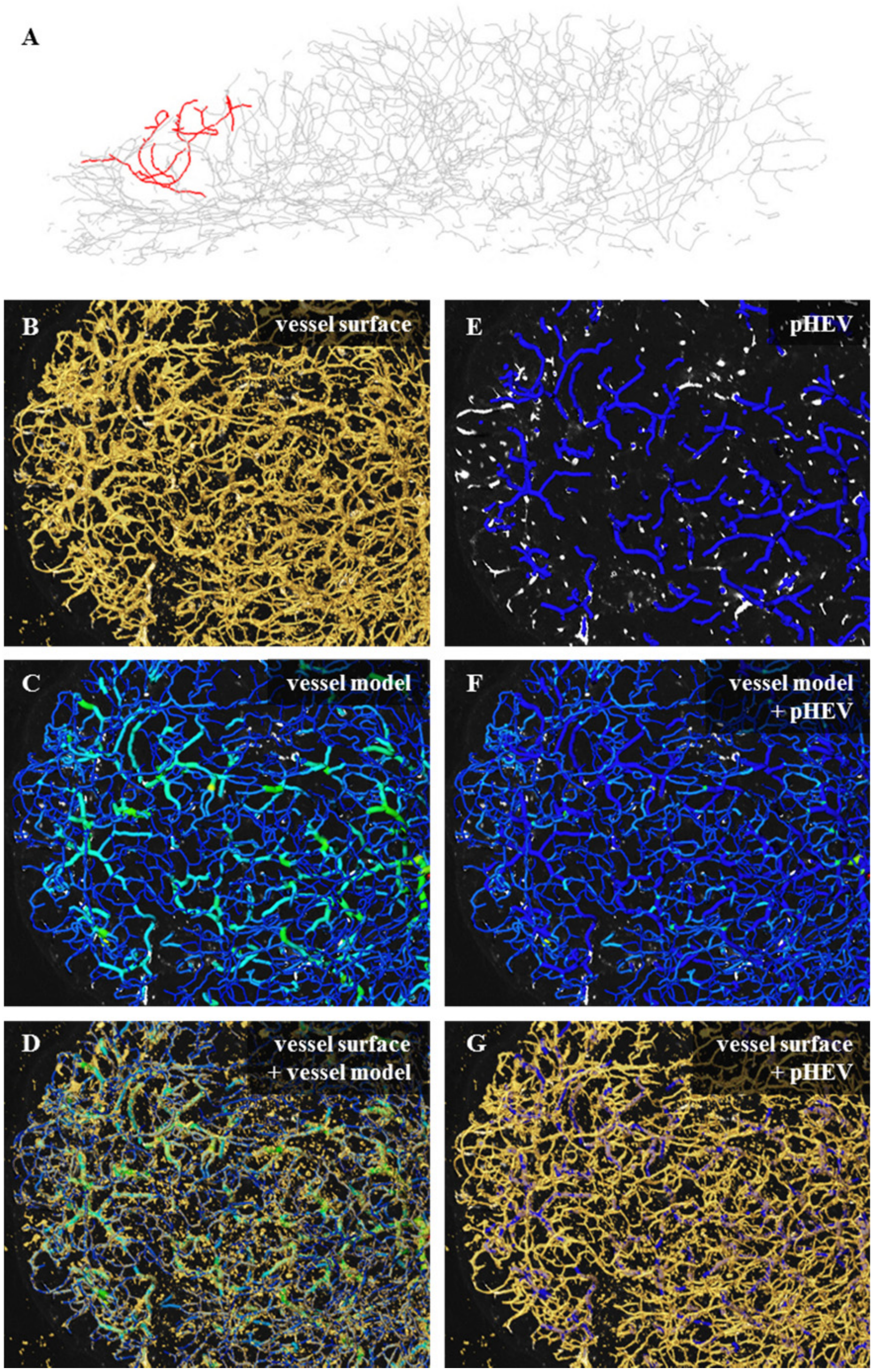

Supplementary Figure 3 │ Extraction of putative HEVs from a 3D blood vessel network.

Vessels with a diameter between 16 and 32 µm were extracted from the 3D vascular network of a mesenteric LN as putative HEVs (pHEV). The extracted pHEVs (grey) form individual branches within the network (red), as can be seen in the 3D filament display in Amira (A). A surface display of the 3D vessel image (B) shows several vessels with 'cobblestone' morphology characteristic for HEVs, which co-localise with postcapillary vessels with dilated dimeters (> 16 µm, green) in the vessel model (D) and with pHEVs (blue, G). In an overlay (F), the extracted pHEVs (E) completely cover all green vessels in the vessel model (C), thereby confirming the selection of HEVs.

Supplementary Note 4: Subregion analysis with different sample region sizes.

T and B cells have distinctive homing regions within LNs, which are each equipped with unique morphological features and subsets of stromal cells to support immune cell function. The B cell follicles contain only few if any blood vessels in the centre and are surrounded by highly vascularised interfollicular regions, while T cell zones show a rather even vascularisation. To compare the blood vessel supply in both regions, we measured the distance to the closest blood vessel within 13 small volume blocks for each region. The size of the blocks was defined by an edge length of either 100 µm, 150 µm, or 200 µm (Supplementary Figure 4). In the T cell area the mean blood vessel distances showed a more or less constant distribution around 28-30 µm, while the data spread was slightly increased towards smaller cube sizes. Interestingly, in the B cell follicles the mean distances to the nearest blood vessel increased significantly from 25 µm to 37 µm with smaller cube sizes alongside an increase in data spread. As a result, the mean distances in the B cell regions were higher than in the T cell regions when 100 µm cubes were investigated, but lower if 200 µm cubes were used (Supplementary Figure 4 a). This relationship signifies the anatomical differences in blood vessel arrangement in these regions. While the T cell zone is more homogenously interspersed with vessels, the inner B cell follicle is rather avascular, but to the outside surrounded by a dense network of vessels. Therefore, the mean blood vessel distances decrease with growing study regions centred in the follicles (Supplementary Figure 4 b). While the 100 µm blocks seem to represent the relatively avascular nature of B cell follicles more clearly, we regard the 200 µm data as more relevant, since it better captures the distance to the nearest supply vessel by incorporating the dense vascular network immediately surrounding the follicle, as well as the vessels within the follicle. (This analysis is also more statistically robust because all volumes analysed contain vessels; in contrast, some 100 µm

blocks lack vessels completely.) Consequently, we proceeded to employ blocks with an edge length of 200 µm to compare vessel features between different subregions from the overall LN vascular network (Figure 7).

A

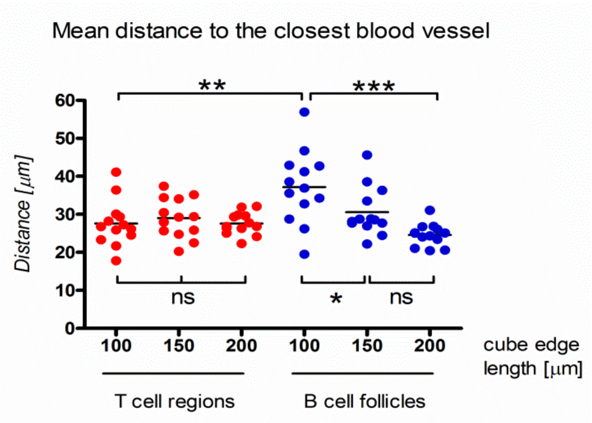

B

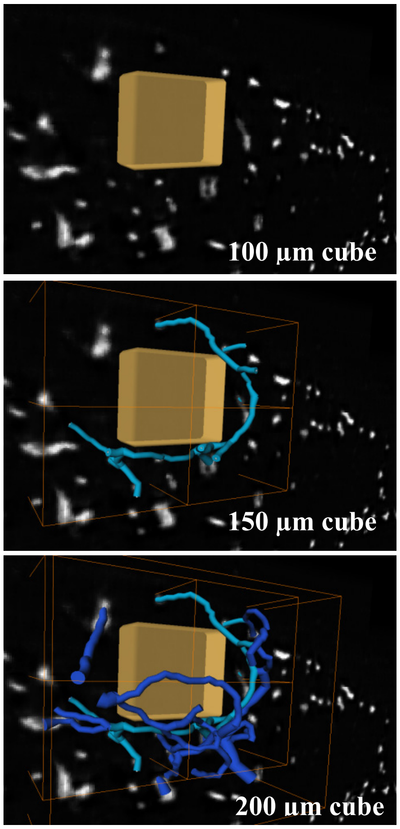

Supplementary Figure 4 │ Effect of varying sample region size on the vessel distance in T and B cell regions.

Within the LN blood vascular network 13 volume blocks with and edge length of 100, 150, and 200 µm were selected from the B cell follicles and T cell zone, respectively. Comparison of the mean distances to the closest blood vessel show significant differences between B cell blocks of different size, while the means in the T cell region remain largely stable (a). The change in vascularisation between small blocks in the centre of a B cell follicle and larger cubes containing more surrounding vessels (blue) was visualised using Amira (b). Data were analysed using 1-way ANOVA, Tukey's comparison (n = 13). ***P<0.001. **P<0.01. *P<0.05. ns = not significant, P>0.05.

Supplementary Movie 1: Side-by-side view of the blood vessel network 3D image and the topology map with colour-coded vessel diameters.

This movie shows the original 3D image data of lymph node vasculature (left) together with the topology map (right) that was generated from the vascular network and used to display the diameter distribution as a rainbow colour spectrum, ranging from large vessels (red) to small vessels (blue).

Supplementary Movie 2: Distribution of diameters in the lymph node vasculature

The diameter distribution within the blood vessel network was sectioned into distinct ranges and overlaid cumulatively. Large vessels (40 – 100 µm, yellow) enter the LN at the hilum, branch into smaller arteries and veins (30 – 40 µm, yellow), that further divide into arterioles and venules (20 – 30 µm, green), to finally reconnect in a bed of small vessels including capillaries (10 - 20 µm, light blue; 5 -10 µm, dark blue).

Supplementary Movie 3: Branching within the lymph node blood vessel network.

A global branch count was performed to identify the shortest path from each node in the blood vessel network to the feeding artery (left) and the collecting vein (right). The distribution of branch counts is visualised as a rainbow colour spectrum with the lowest branch counts appearing blue and the highest branch counts (>30) shown in red.

Supplementary Movie 4: Specialised vessels and detection of voids

The arterial (red) and venous (blue) branches containing vessels of diameters > 15 µm were superimposed with the full LN blood vessel network model (grey). Visualisation of pHEVs (magenta)

illustrates their location in between both branches and small voids with distances > 60 µm to a neighbouring vessel can be found interspersed within the network.

Supplementary Movie 5: Location of predicted functional subregions within the lymph node vasculature

Selected subregions including B cell follicles (blue), predicted T cell regions (dark red), the hilum (yellow), and regions with dense vasculature (green) are displayed within the vascular network model and overlaid with avascular regions (voids, bright red).

Supplementary References

1 Ibanez, L. & Schroeder, W. *ITK Software Guide*. (Kitware, Inc., 2005).

2 Yoo, T. S. *et al.* Engineering and algorithm design for an image processing Api: a technical report on ITK--the Insight Toolkit. *Stud. Health Technol. Inform.* **85**, 586-592 (2002).

3 Lee, T. C., Kashyap, R. L. & Chu, C. N. Building Skeleton Models Via 3-D Medial Surface Axis Thinning Algorithms. *Cvgip-Graph. Model. Im.* **56**, 462-478 (1994).

4 Homann, H. Implementation of a 3D thinning algorithm. *Insight J.*, http://hdl.handle.net/1926/1292 (2007).

5 Dijkstra, E. W. A Note on Two Problems in Connexion with Graphs. *Numer. Math.* **1**, 269-271 (1959).

6 Beare, R. Morphology with parabolic structuring elements. *Insight J.*, http://hdl.handle.net/1926/1370 (2008).

7 Lloyd, C. M., Phillips, A. R., Cooper, G. J. & Dunbar, P. R. Three-colour fluorescence immunohistochemistry reveals the diversity of cells staining for macrophage markers in murine spleen and liver. *J. Immunol. Methods* **334**, 70-81, doi:10.1016/j.jim.2008.02.005 (2008).
